# Is independence of older adults safe considering the risk of falls?

**DOI:** 10.1186/s12877-017-0461-0

**Published:** 2017-03-14

**Authors:** Dorota Talarska, Magdalena Strugała, Marlena Szewczyczak, Sławomir Tobis, Michał Michalak, Izabela Wróblewska, Katarzyna Wieczorowska – Tobis

**Affiliations:** 1 Department of Preventive Medicine, University of Medical Sciences, Święcickiego 6, 60-179 Poznań, Poland; 2 Department of Palliative Care, University of Medical Sciences, Laboratory of Geriatric Medicine, Poznań, Poland; 3 Department of Geriatrics and Gerontology, University of Medical Sciences, Laboratory of Occupational Therapy, Poznań, Poland; 4 Department of Computer Science and Statistics, University of Medical Sciences, Poznań, Poland; 5 Faculty of Health Sciences, Wroclaw Medical University, Wrocław, Poland

**Keywords:** Falls, Elderly people, Life activities

## Abstract

**Background:**

Falls affect approx. 30% of elderly population per year. They cause major injuries and reduce independence of the older adults’ functioning.

*The main objective* of the study was to evaluate the degree of independence and find the fall risk factors in the study group.

**Methods:**

The study included 506 – older adults. The study group included patients from GP clinics and members of two senior centers. The study duration was 12 months. Our study tools included EASY- Care Standard 2010 questionnaire, Abbreviated Mental Test Score (AMTS), Index Barthel, Instrumental Activities of Daily Living Scale (IADL), Geriatric Depression Scale (GDS), Timed Up and Go (TUG).

**Results:**

The study included 357 (70.6%) female and 149 (29.4%) male subjects. The mean age of the study group patients was 75.7 years ± 8.0. Most of the older adult subjects were independent in both basic (Index Barthel) and instrumental (IADL) activities. Gait fluency evaluated in TUG scale found slow and unsteady gait in 33.7% of the subjects. 27.5% of the subjects used mobility aids when walking. In the Risk of falls scale, 131 subjects (25.89%) were at risk of falls. According to logistic regression the main risk of fall determinants (*p* <0.05) in the study group were: age, previous falls, feet problems, lack of regular care, impaired vision, urinary incontinence, pain, sleeping disorders, and lowered mood.

**Conclusions:**

Risk of falls increases in people less independent in terms of basic and complex life activities and in people with depression. Most of the risk factors can be modified. It is necessary to develop a standard procedure aimed at preventing falls in the elderly.

## What is known on the issue

1. Falls affect approx. 1/3 of older adults population per year.
2. They significantly reduce independence of the elderly people’s functioning.
3. Falls generate costs related to the need of support.
4. Risk of falls factors previously confirmed by research studies include: advanced age; history of falls; decrease in physical sensory and cognitive activity; impaired balance and gait; sphincter function disorder; sleep and mood disorders; pain complaints; and medications.

## What this paper adds

1. The factors found have been confirmed to be modifiable and that fall prevention strategy should be based on them.
2. The fact that risk of falls increases with age indicates that all older adults require supervision regardless of their health.
3. EASY – Care Standard 2010 questionnaire helped identify the limitations in independent functioning of older adults and factors for risk of falls disability, and round-the-clock care.
4. Once the intensity of a given risk factor is determined it will be possible to establish priorities in risk prevention and in support planning.
5. In addition to this other risk factors for falls have been found, which had frequently been ignored or not associated with falls, such as the feeling of loneliness or hearing impairment.

## Background

Frequency of falls of older adults is difficult to determine because incidents with no major injuries are rarely reported. It is assumed that approx. 30% of people above the age of 65 suffer from a fall every year [1–3]. Consequences vary, they may include minor injuries but also frequently result in deterioration of quality of life or death [4, 5]. High interest in falls of the older adults arises primarily from the fact that the fall causes a rapid change in health and, thus, the need to provide them with care, frequently round the clock. Expenditure of medical and social services arising from falls of older adults, in particular costs of: hospital treatment, out-patient procedures, rehabilitation, and the need to provide a permanent carer following discharge home, are significant [3]. Therefore, care providers undertake various measures in order to reduce these expenses. Prevention relies on identification of fall factors for a specific person and reduction of modifiable risk factors, health supervision, and gait fluency improvement [5–8].

The most frequently encountered risk factors include: impaired balance and gait, lower muscle strength, lower psychomotor ability, polytherapy, and therapy with benzodiazepines [6, 9, 10]. Additional, health-related factors include: pain; sense of weakness; chronic diseases – particularly neurological diseases, heart diseases, and diabetes; cognitive disorders, and urinary incontinence [4, 5, 9, 11]. Demographic factors discussed by authors of earlier research papers include: elderly age, female sex, low sociodemographic status [4]. Further functioning of persons who had previously suffered from falls depends on the consequences of fall, experience learned, and the degree of fear of falling again [12, 13]. Time of fall is also a noteworthy aspect, especially the time of the day, due to the morning or evening sleepiness and fatigue. Some authors report that falls suffered by people staying at their place of residence would usually take place in the morning and in the evening [6] while others it was during house chores [7, 14]. Scales most frequently used to assess gait fluency include Time Up and Go test (TUG) as well as Berg Balance Scale (BBS), and Tinneti test [6, 15]. Aachen Falls Prevention Scale is also being increasingly frequently referred to as a useful tool in independent fall risk monitoring by older adults themselves [6].

Due to medical and social consequences, falls are a field of interest of various care providers. Many countries undertake measures to develop a standard procedure. Poland, due to the need to combine activities of two sectors, medical care and social welfare, is still searching for the best strategy of action.

The main objective of the present study was to:

1. evaluate the degree of independence of older adults;
2. determine the effect of functional condition, demographic and social factors on the risk of falls in the study group;
3. identify the factors to be taken into consideration when planning falls prevention in the study group;
4. prove suitability of the EASY-Care Standard 2010 questionnaire in evaluation of the risk of falls.

## Method

### Study organisation

The study has been conducted for 12 months in two GP clinics and two senior centers. Both facilities accepted our students for traineeships. The collected questionnaires helped them learn more of the barriers in independent functioning of older adults. Over this time, the study included 532 older adults. 20 senior citizens withdrew from the study, and 6 questionnaires were not completely filled in and, as such, they were not included in the analysis. Eventually, the analysis was based on 506 questionnaires.

Prior to filling in the questionnaire, the researcher would have explained the study and discussed the tools used. Senior citizens could use the researchers assistance when filling in the questionnaire or withdraw from the study at any time throughout its duration.

Each test started with an evaluation of the person’s cognitive abilities using AMTS scale, and only persons with a score of at least 6 were included in further stages of the study.

*Inclusion criteria:*

- age: ≥60,
- mental ability allowing filling in the questionnaire: Abbreviated Mental Test Score (AMTS) > 6,
- resident of Poznań or its vicinity,
- consent to participation in the study.

### Study tools

1. Abbreviated Mental Test Score (AMTS) was used to evaluate cognitive abilities [16]. Scoring range in this scale was from 0 to 10 points. A score of 4–5 meant a moderate mental impairment and 3–0 – a severe mental impairment. Normal mental abilities were within the score range from 6 to 10.
2. The degree of independence in functioning was analyzed using EASY-Care Standard 2010 which allows a comprehensive functional evaluation and sociomedical needs of elderly people living in their own homes and those residing in care institutions [17–20]. It analyses 7 areas of functioning: 1. Seeing, hearing and communicating, 2. Looking after yourself, 3. Getting around, 4. Your safety, 5. Your accommodation and finance, 6. Staying healthy, 7. Mental health and well-being.
  3 scales in the final section of the questionnaire summarize the results:
  Independence score – to evaluate independence of the study subject in terms of basic and complex life activities. Scoring range is 0–100. The higher the score, the greater the dependence on other people.
  Risk of breakdown in care – is a scale used to identify the risk of 24/7 care. Scoring range is 0–12. The risk of continuous care increases with higher score.
  Risk of falls – a scale allowing assessment of the risk of falls. Final score is from 0 to 8 points. 3 or more points is understood as risk of falls. EASY-Care Standard 2010 tool has been subject to psychometric evaluation during previous studies [19, 21].
3. Geriatric Depression Scale (GDS) – used for Comprehensive Geriatric Assessment [22]. It helps diagnose depression in elderly people. Scoring range is 0–15.
  Score of 0–5 is no depression, 6–10 is moderate depression, and 11–15 – severe depression.
4. Barthel Index of Activities of Daily Living (Index Barthel) – a scale of 0–100 points. Patients with score above 86 are in good functional condition, while score below 20 means severe impairment [23].
5. Instrumental Activities of Daily Living Scale (IADL) – the version with 9 questions, with 3 options of answers was used: 1 point means full dependence, 2 points – partial dependence, and 3 – independence. Maximum total score is 27 points [24].
6. Timed Up and Go (TUG) is a gait fluency assessment on a distance of 3 m. It measures the time of: getting up from a chair, walking a distance of 3 m, turning by 180^o^, return and sitting again. Older adults do this test twice, and the better result is then analyzed. Risk of falls occurs if more than 12 s are required to complete these tasks [15]. For statistical purposes, the following classification was adopted: steady and fast gait, unsteady and fast gait, steady and slow gait, unsteady and slow gait.

#### Statistical analysis

Analysis was performed using STATISTICA 10 PL package (StatSoft Inc.). Differences between two independent groups were evaluated using non-parametric Mann-Whitney test. Correlations were assessed using Spearman’s r_s_ rank correlation coefficient. Nominal variables were analyzed using chi-squared test for independence. To identify factors significantly affecting the risk of falls, logistic regression model was used. Results obtained were presented as odds ratios with 95% confidence intervals. All the tests were considered statistically significant at *p* <0.05.

A stepwise multiple logistic regression with backward elimination was also performed.

### Study group characteristics

The study included 506 subjects aged above 60 (60–101 years). Of these, 357 subjects (70.6%) were female and 149 (29.4%) male. The mean age of the study group patients was 75.7 years ± 8.0. Most of the subjects resided with their family (51.4%) or spouse (25.7%).

## Results

The study started with evaluation of participants’ cognitive abilities using AMTS screening tool. Average score in the group was 9.2 ± 1.0 – 9.3 ± 1.0 for women and 9.1 ± 1.0 for men (Mann-Whitney test, p = 0.2691). In terms of basic activities of daily living (Barthel Index), the subjects scored from 50 to 100, with median score of 95. Severe impairment was found in nearly 25% of the group. In terms of complex activities of daily living (IADL), the subjects would score from 11 to 27 (with median score of 25). GDS found 37.6% of the group to have moderate and 9% severe depression. 52.5% had no depression. Gait fluency evaluated in TUG scale found slow and unsteady gait in 33.7% of the subjects. 27.5% of the subjects used mobility aids when walking, mostly walking canes and crutches (21.8%). 6% of the elderly subjects used the assistance of another person.

Functioning in the study group was assessed using EASY-Care Standard 2010 questionnaire recognised as a good tool to evaluate the need of assistance [19, 21]. Of the 7 analyzed areas, the greatest need of assistance was found in: Mental health and well-being (100%), Staying healthy (99.0%), Getting around (63.0%), Seeing, hearing and communicating (47.5%).

EASY-Care Standard 2010 questionnaire is summarized by 3 risk scales. Independence Score (0–100) of the study group was on average 14.4 ± 19.7, and average risk of breakdown (0–12 points) was 3.4 ± 2.5. In the Risk of falls scale, 131 subjects (25.89%) were at risk of falls.

Elderly subjects were taking from 0 to 12 medicines, on average 4.2 ± 2.9 per day.

### Risk of falls – statistical analysis

In the group of subjects prone to falls, difficulties in going out, feeling unsafe outside home, and vision impairment were the dominant factors in EASY-Care Standard 2010 risk of falls scale. 74.81% of people prone to falls and 21.07% not prone to falls experienced previous falls Table 1.

**Table 1:** Comparison of risk of fall factors in the Risk of falls scale

Risk of falls	Subjects prone to falls		Subjects not prone to falls		Chi-Square test *P-*value	
	*N*-131	25.89%	*N*-375	74.11%		
Seeing problems	91	69.47	112	29.87	66.68	*p* <0.001
Problems in getting around	34	25.95	2	0.54	94.94	*p* <0.001
Feet problems	71	54.20	57	15.20	78.14	*p* <0.001
Previous falls	98	74.81	79	21.07	139.74	*p* <0.001
Difficulties in going out	103	78.63	39	7.71	233.47	*p* <0.001
Feeling unsafe at home	13	9.92	8	2.13	14.81	*p* <0.001
Feeling unsafe out of home	92	70.23	26	6.93	217.51	*p* <0.001
Excessive drinking	6	4.58	7	1.87	2.86	*p* = 0.091

The study group were found to have a negative correlation between the risk of falls scale in EASY-Care Standard 2010 and AMTS score (r_s_ -0.1696 *p* = 0.007) and a positive correlation with GDS (r_s_ 0.2347 *p* <0.001). Analysis shows that the risk of falls increases with poorer mental ability (lower AMTS score) and depression (higher GDS score). A negative correlation (r_s_ test) was also found between level of functioning in basic activities of daily living (*p* <0.001) and complex activities (*p* <0.001) and the risk of falls. The risk of falls increased with poorer functioning in both scales.

Analysis of individual EASY-Care Standard 2010 fields found the activities causing the greatest difficulties in independent functioning, and then identified those which might be the cause of falls and were not included in the Risk of falls scale Table 2.

**Table 2:** Additional factors contributing to falls

Activities	Risk of falls – 131		No risk of falls – 375		*P-*value*
	*N*	%	*N*	%	*p* <0.001
Hearing					
difficulties	57	43.51%	103	27.47%	*p* <0.001
no difficulties	74	56.49%	272	72.53%	
Using bathtub					
difficulties	83	63.36%	33	8.80%	*p* <0.001
no difficulties	48	36.64%	342	91.20%	
Urinary incontinence					
difficulties	94	71.76%	122	32.53%	*p* <0.001
no difficulties	37	28.24%	253	67.47%	
Feet problems					
yes	71	54.20%	57	15.20%	*p* <0.001
No	60	45.80%	318	84.80%	
Walking stairs					
difficulties	94	71.76%	42	11.20%	*p* <0.001
no difficulties	37	28.24%	333	88.80%	
Regular exercise					
yes	39	29.77%	280	74.67%	*p* <0.001
no	92	70.23%	95	25.33%	
Feeling lonely					
yes	78	59.54%	154	41.07%	*p* <0.001
no	53	40.46%	221	58.93%	
Sleeping problems					
yes	93	70.99%	205	54.67%	*p* = 0.001
no	38	29.01%	170	45.33%	
Pain					
yes	105	80.15%	240	64.00%	*p* <0.001
no	26	19.85%	135	36.00%	
Depression					
yes	73	55.73%	102	27.20%	*p* <0.001
no	58	44.27%	273	72.80%	
Forgetfulness					
yes	74	56.49%	131	34.93%	*p* <0.001
no	57	43.51%	244	65.07%	

* Chi-Square test

As a part of risk of falls factors, the following demographic variables were analyzed: sex, age, and form of residence. Relationship with age (chi^2^ *p* <0.001) has been found but no relationship with sex (chi^2^ *p* = 0.215) or from of residence (chi^2^ *p* = 0.803).

Logistic regression was performed for the factors which created most difficulties (over 30%) to the older adults. Table 3.

**Table 3:** Logistic regression taking into consideration selected variables

Variables	OR	95% CL
Age	4.4	2.72–6.97
Previous falls	11.1	6.97–17.75
Feet problems	6.6	4.23–10.01
No regular exercise	6.8	3.94–12.39
Impaired vision	4.9	2.82–8.45
Urinary incontinence	3.5	1.99–6.15
Pain	2.3	1.41–3.67
Sleeping problems	2.0	1.33–3.13
Depression	3.3	2.22–5.0
Forgetfulness	2.4	1.61–3.63

Multiple logistic regression has found the following to be significant risk of falls factors: no regular exercise (OR 7.0; 95% CL 3.94-12.39), sight problems (OR 4.8; 95% CL 2.82- 8.45), and urinary incontinence (OR 3.4; 95% CL 1.99-6.15).

## Discussion

The main objective of the present study was to evaluate the degree of independence and find the fall risk factors in the study group. Authors found that although the study group functioned well in terms of activities of daily life, nearly everyone was affected by at least 1 - risk factor due to diversity of risk factors. Larger number of risk factors significantly increases the probability of falling which is why it is important to reduce each of these factors [6]. In our study, risk factors for falls were identified, some of which are modifiable i.e. could be reduced or their adverse effect mitigated. These include: vision impairment, hearing impairment, feet problems, urinary incontinence, little exercise, and lowered mood. Lack of standard procedure addressed at preventing the risk of falls in Poland leads to injuries in many people over 60 where such injuries could have been avoided. The objective of the present study was to evaluate the degree of independence of elderly people and find the fall risk factors in the study groups which should be considered when organizing care. Identification of the most predisposing factors helps organize adequate support focused on preventing falls. Age strongly affects the risk of falls, as the risk increases with age. In the study group, risk of falls was four times higher in patients aged over 75. The data obtained are similar to those published previously [4]. Age is a variable not subject to modification, therefore programs should be put in place including all older adults as potentially prone to falls. Sex is not really significant. Some authors point out that women living alone and obese are more prone to falls [4]. Although our study has not confirmed such a correlation, females were slightly more numerous in the group of people prone to falls.

Measures addressed at the older adults patient and his or her closest family is an important aspect of preventing falls at home [18]. Vision improvement by cataract removal or choice of glasses significantly reduces the number of falls. In addition to this, as shown by Gillespie et al., wearing non-slip shoes or elimination of podiatric problems also reduces the number of incidents. Regular exercise, including tai chi, or using mobility aids such as canes also have a positive effect [1, 4, 10, 25, 26]. Walking is recommended for falls prevention. Evidence suggests, however, that, walking can increase risk of falls, thus walking alone should not be recommended as a fall prevention strategy [7]. Balancing exercise, and tailored workouts improving cardiovascular and respiratory efficiency and muscle strength are particularly recommended [8, 10].

Consequences of a fall include post-fall syndrome characterized by fear of getting around and falling again [13]. Older adults frequently describe falls as a negative experience associated with the fear of being dependent on others and the need to leave their home due to reduced independence [12]. In our study, approx. 1/3 of the group had previous falls. They should be provided with particular support as the history of falling frequently causes fear of undertaking activities of daily life [13].

Frailty syndrome and gait speed are also good indicators of the risk of falls [2, 6, 25]. Various scales facilitating analysis of gait changes and measurement of muscle strength are being implemented to monitor such indicators [6, 8]. Our study only took into consideration gait fluency measured using Timed Up and Go test. Subjects from the risk group walked slowly and unsteadily.

Stabilization of health problems, in particular chronic diseases and mood disorders, is also an important aspect [9, 11]. Elimination of pain is important in prevention of falls. In terms of pain, it works two ways; on one hand, pain may reduce the mobility range, on the other – little amount of exercise may reduce gait fluency and increase pain [27]. Pain also intensifies depression symptoms [27]. In the study group, pain increased risk of falls by two times.

Precise identification of the risk group and modification of risk factors is of crucial importance in preventing falls [4, 7, 10]. Especially so that approx. 70% of the elderly patients had not consulted a physician following a fall, as shown by Qin Z. and Baccaglini L. [4]. This may be the sign of smaller injuries but also unawareness of consequences. Optimum prevention methods should, therefore, be based on multi-sector actions [5, 10]. These actions should be based on evaluation of mental and physical condition, analysis of functioning abilities of the elderly, and introduction of gait improving exercises. Further actions include evaluation of home environment in terms of the risk of fall and introduction of improvements. Care needs may be evaluated using EASY-Care standard 2010 questionnaire [17–20, 28]. Especially as it allows full functional evaluation of the elderly patients. It shows that the risk of falls may be associated with problems indirectly related to physical exercise, such as calluses, feet deformation, or urinary incontinence. Education should be an important concurrent activity [4, 5]. Older adults people frequently undertake activities not giving consideration to safety of performing them. They do not take into account their present health or mental and physical abilities. Hanging curtains, taking things out of low shelves may contribute to falls with increasing age and older adults should be aware of that. The best effects of preventive measures are obtained with multi-sectoral activities focused on eliminating both external and internal factors [7, 29]. Such effects are possible when healthcare professionals co-operate with welfare workers and with active involvement of the elderly patient and their family. Regular assessments of biopsychosocial functioning of the elderly people, allowing identification of reduced independence and degree of compliance with recommendations, are also of utmost importance. They help tailor the support to individual patients. It is also extremely important to devise a program of support for the older adults, focused on risk of falls factors, comprising various assistance services.

## Conclusions

Risk of falls increases in people less independent in terms of basic and complex life activities and in people with depression. Most of the risk factors can be modified. It is necessary to develop a standard procedure aimed at preventing falls in the older adults.

## Abbreviations

AMTS: Abbreviated mental test score
EASY- Care: EASY- care standard 2010 questionnaire
GDS: Geriatric depression scale
GP: General practitioner
IADL: Instrumental activities of daily living scale
Index Barthel: Barthel index of activities of daily living
TUG: Timed up and go.
